# Pruritus as the First Clue: Early Diagnostic Workup Uncovering an Ampullary Adenoma

**DOI:** 10.7759/cureus.110135

**Published:** 2026-06-02

**Authors:** Lara Calegari, Andrew Korman, Sahil Raval, Archit Garg, Ricardo Poroski

**Affiliations:** 1 Internal Medicine, Saint Peter's University Hospital/Rutgers Health Robert Wood Johnson Medical School, New Brunswick, USA; 2 Gastroenterology and Hepatology, Saint Peter's University Hospital, New Brunswick, USA; 3 Gastroenterology and Hepatology, Saint Peter's University Hospital/Rutgers Health Robert Wood Johnson Medical School, New Brunswick, USA; 4 Internal Medicine, Saint Peter's University Hospital, New Brunswick, USA; 5 Surgical Oncology, Hospital Nossa Senhora dos Prazeres, Lages, BRA

**Keywords:** ampullary adenoma, endoscopic papillectomy (ampullectomy), high-grade dysplasia, jaundice cholestatic, malignant transformation

## Abstract

Ampullary adenomas are uncommon lesions with malignant potential and may present with nonspecific symptoms, making diagnosis challenging. This is the case of a 64-year-old woman who presented with diffuse pruritus, right upper quadrant discomfort, and a cholestatic liver pattern injury, posteriorly found to have an ampullary lesion causing biliary obstruction. Initial endoscopic evaluation and biopsy suggested low-grade dysplasia, with temporary improvement after biliary decompression. However, persistent symptoms prompted repeat intervention with endoscopic papillectomy, and final pathology demonstrated high-grade dysplasia with positive margins, raising concern for an underlying malignancy not identified on the initial biopsy. This case highlights the variable clinical presentation of ampullary adenomas and emphasizes the limitations of superficial tissue sampling, underscoring the importance of complete histologic evaluation and close follow-up in suspicious ampullary lesions.

## Introduction

Ampullary adenomas are glandular polyps that originate in the ampullary complex of the duodenum, close to the convergence of the common bile duct (CBD) and pancreatic duct (PD). These lesions might arise from different structures such as the pancreas, CBD, duodenum, or the sphincter of Oddi complex (which includes the sphincter of Oddi and papilla of Vater) [1].

The incidence of ampullary neoplastic lesions (ANLs), originating from the papilla of the Vater, is considerably low and constitutes approximately 0.6-0.8% of all gastrointestinal tumors. Out of these ANLs, 0.04-0.12% are considered ampullary adenomas after post-mortem autopsy analysis [2].

Ampullary adenomas are mostly found in patients aged above 40 years of age, with a high prevalence being detected in the sixth to seventh decades of life. Factors associated with a higher risk of malignant transformation include a size > 10 mm (diameter) or villous changes on histopathology. The likelihood of malignant foci in such cases is as high as 50% [2]. Due to the complex location of these ampullary adenomas, which makes their detection challenging and a potential risk for malignant transformation, early detection, precise staging, and management are necessary.

This case is important to report given its atypical presentation, where diffuse pruritus was a key clinical feature prompting evaluation rather than classic symptoms. It underscores the diagnostic challenges, the dynamic clinical evolution, and the stepwise workup that ultimately led to the identification of an ampullary lesion, which, on definitive evaluation and ampullectomy, was found to be of a higher grade than initially suspected.

## Case presentation

A 64-year-old woman presented to the hospital with a four-day history of right upper quadrant (RUQ) abdominal discomfort. It was associated with diffuse pruritus and subjective fevers. She had a past medical history of hypertension, type 2 diabetes, hyperlipidemia, congestive heart failure, chronic obstructive pulmonary disease with oxygen dependence, chronic kidney disease, osteoporosis, anemia of chronic disease, and chronic pulmonary embolism (on anticoagulation). Her family history was significant for alcohol-related liver disease in her mother and brother. The patient reported consuming large amounts of alcohol herself for 10 years, but she had discontinued this 30 years ago.

On examination, the patient was afebrile and hemodynamically stable. Physical exam was significant for mild subjective icterus of the sclera and skin, which got progressively worse during the hospitalization course, associated with diffuse abdominal discomfort more pronounced at the RUQ and epigastric area. Laboratory was consistent with cholestatic liver injury, with significantly elevated alkaline phosphatase (ALP) and gamma-glutamyl transferase (GGT), elevated total bilirubin, aspartate aminotransferase (AST), alanine transaminase (ALT), with normal international normalised ratio (INR) and platelets. Although the patient reported a remote history of alcohol use, this was not considered contributory in view of the cholestatic presentation.

Urine toxicology, serum salicylate, and serum acetaminophen levels were negative. Given elevated liver enzymes, a workup for liver injury was ordered, including serum hemochromatosis DNA mutation, ceruloplasmin, α-1 antitrypsin, smooth muscle antibody, and anti-mitochondrial antibody, antineutrophil cytoplasmic antibodies (ANCA), antinuclear antibody (ANA), which were negative, and immunoglobulins (mildly elevated IgA). The viral panel was negative for hepatitis C, cytomegalovirus, and human immunodeficiency virus. Epstein-Barr virus and herpes simplex virus IgG were positive, and the patient had immunity to hepatitis A and hepatitis B. Quantitative laboratory investigations obtained during the diagnostic workup are summarized in Table 1. 

**Table 1:** Additional laboratory and serologic investigations Laboratory evaluation performed during the workup of abnormal liver enzymes and alternative causes of hepatic injury. CMV: cytomegalovirus; HSV: herpes simplex virus

Test	Patient Value	Reference Range
α-1 antitrypsin	185	83-199 mg/dL
Ceruloplasmin	37	14-48 mg/dL
IgA	619	70-400 mg/dL
IgM	55	40-230 mg/dL
IgG	1547	700-1600 mg/dL
HSV1/2 IgG	55.2 / 13	<0.90 Negative; 0.90-1.09 Equivocal; >1.09 Positive
CMV IgM	< 30	>35 AU/mL
Serum acetaminophen/salicylate	<10	10-30 mcg/mL
Salicylate levels	<1	2-20 mg/dL

**Figure 1:**
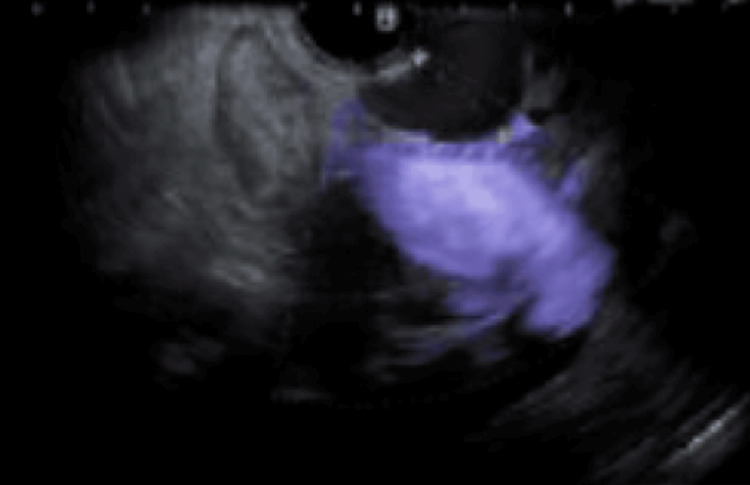
Endoscopic ultrasound demonstrating a hypoechoic ampullary mass arising at the level of the major papilla, measuring approximately 22.9 × 10 mm. The lesion appears confined to the mucosal/superficial layer without clear evidence of deep invasion into adjacent structures.

**Figure 2:**
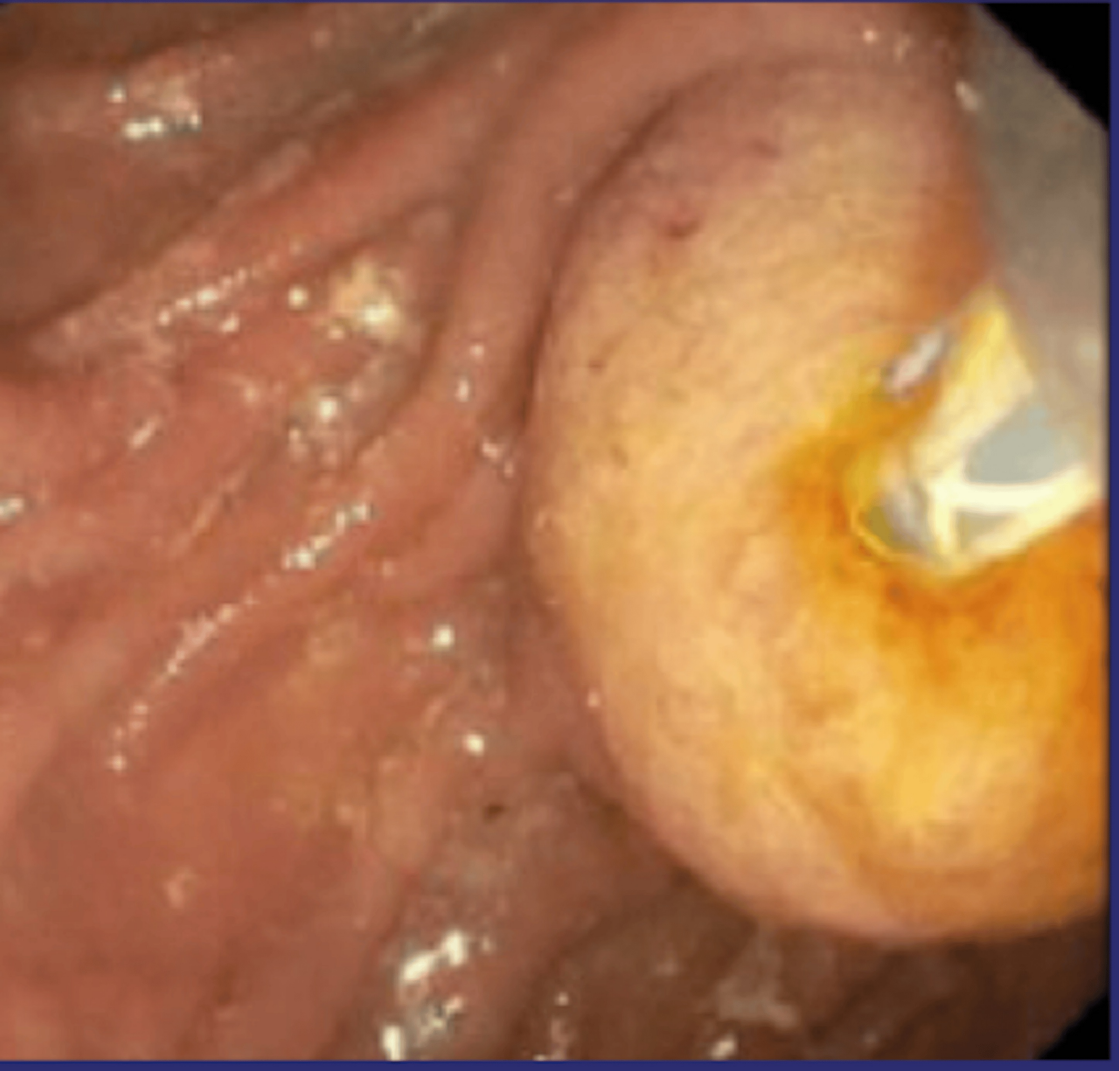
ERCP demonstrating ampullary adenoma involving the major papilla The ampulla appears enlarged and nodular. ERCP: endoscopic retrograde cholangiopancreatography

RUQ ultrasound showed post-cholecystectomy status with a 9-mm CBD. Subsequent magnetic resonance cholangiopancreatography (MRCP) demonstrated extrahepatic ductal dilation up to 12 mm without any evidence of choledocholithiasis and multiple ≤7-mm pancreatic cysts. This was followed by an endoscopic ultrasound (EUS) for better evaluation. The EUS revealed an ampullary mass with CBD dilation up to 10 mm (Figure 1). EUS was followed by endoscopic retrograde cholangiopancreatography (ERCP) in which bile-duct brushings were obtained in the lower third of the main bile duct, and ampullary biopsies were taken (Ampullary adenoma is shown in Figure 2).

**Figure 3:**
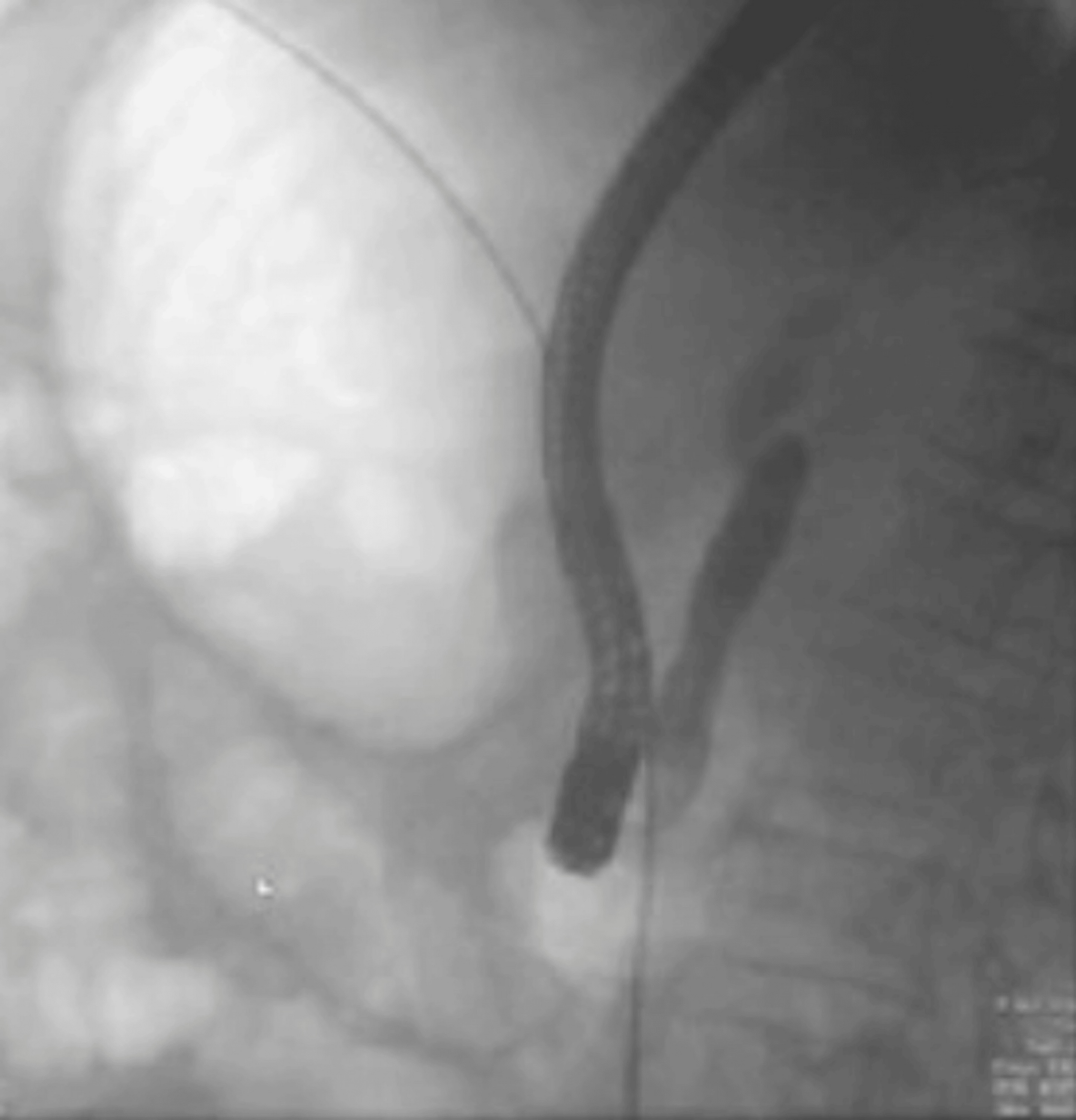
ERCP fluoroscopic imaging demonstrating CBD stent placement Cannulation of the CBD was achieved endoscopically for diagnostic evaluation and therapeutic intervention. ERCP: endoscopic retrograde cholangiopancreatography; CBD: common bile duct

A plastic CBD stent (7Fr, 7cm) was placed into the CBD duct during the procedure (Figure 3). Bile flowed through the stent.

Following the intervention, the patient demonstrated improvement in bilirubin levels and resolution of jaundice. A summary of laboratory trends during the hospitalization course is provided in Table 2. Pathology confirmed an ampullary adenoma with low-grade dysplasia.

**Table 2:** Serial liver function tests during clinical course ALP: alkaline phosphatase; AST: aspartate aminotransferase; ALT: alanine aminotransferase; INR: international normalized ratio “-” indicates values not obtained at that time point.

Parameters	Day 1 of hospitalization	Day 5 of hospitalization	Discharge (Day 7)	Reference Range
ALP	1962	1533	1394	56-119 U/L
AST	140	61	42	17-59 U/L
ALT	170	79	64	0-50 U/L
Total Bilirubin	3	9.2	2.7	0.1-1.2 mg/dL
Bilirubin Direct	1.4	6.5	-	0.0-0.3 mg/dL
Bilirubin Indirect	0.3	-	-	0.0-1.1 mg/dL
Platelet	461	-	365	150-400 x 10^3^/cumm
INR	1.08	-	-	0.89-1.11

**Figure 4:**
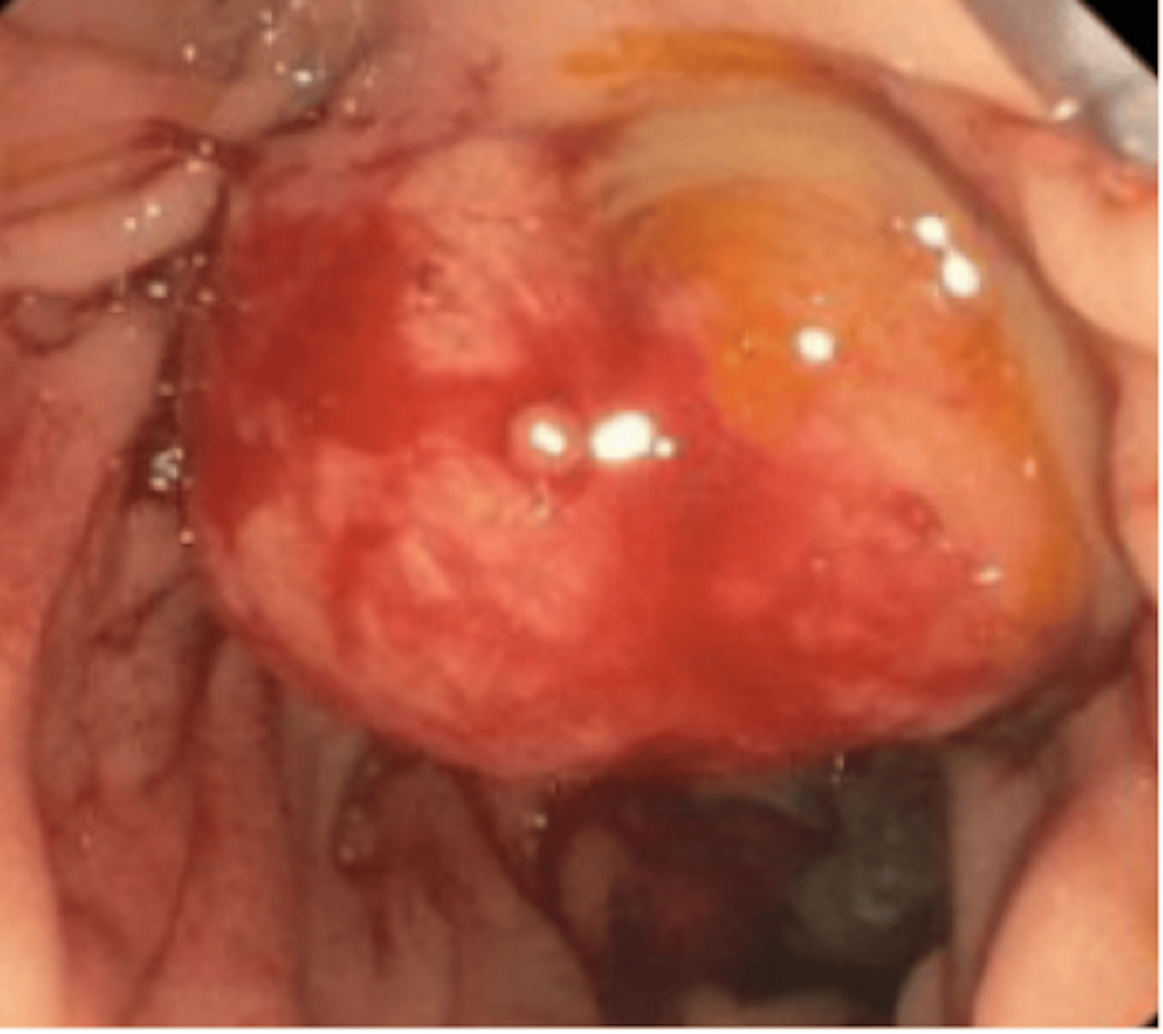
Repeat ERCP demonstrating friable ampullary lesion with surface bleeding Repeat ERCP perfomed approximately four months after the initial presentation demonstrates a ampullary lesion with erythematous and irregular mucosal appearance. The lesion appeared more prominent compared to prior examination with areas of superficial bleeding noted over the lesion surface. ERCP: endoscopic retrograde cholangiopancreatography

Approximately four months later, the patient re-presented with persistent abdominal pain, prompting further evaluation. ERCP demonstrated mild pancreatic duct dilation and choledocholithiasis, with complete stone removal via balloon extraction. The persistent ampullary lesion visualized during this repeat ERCP is shown in Figure 4. 

**Figure 5:**
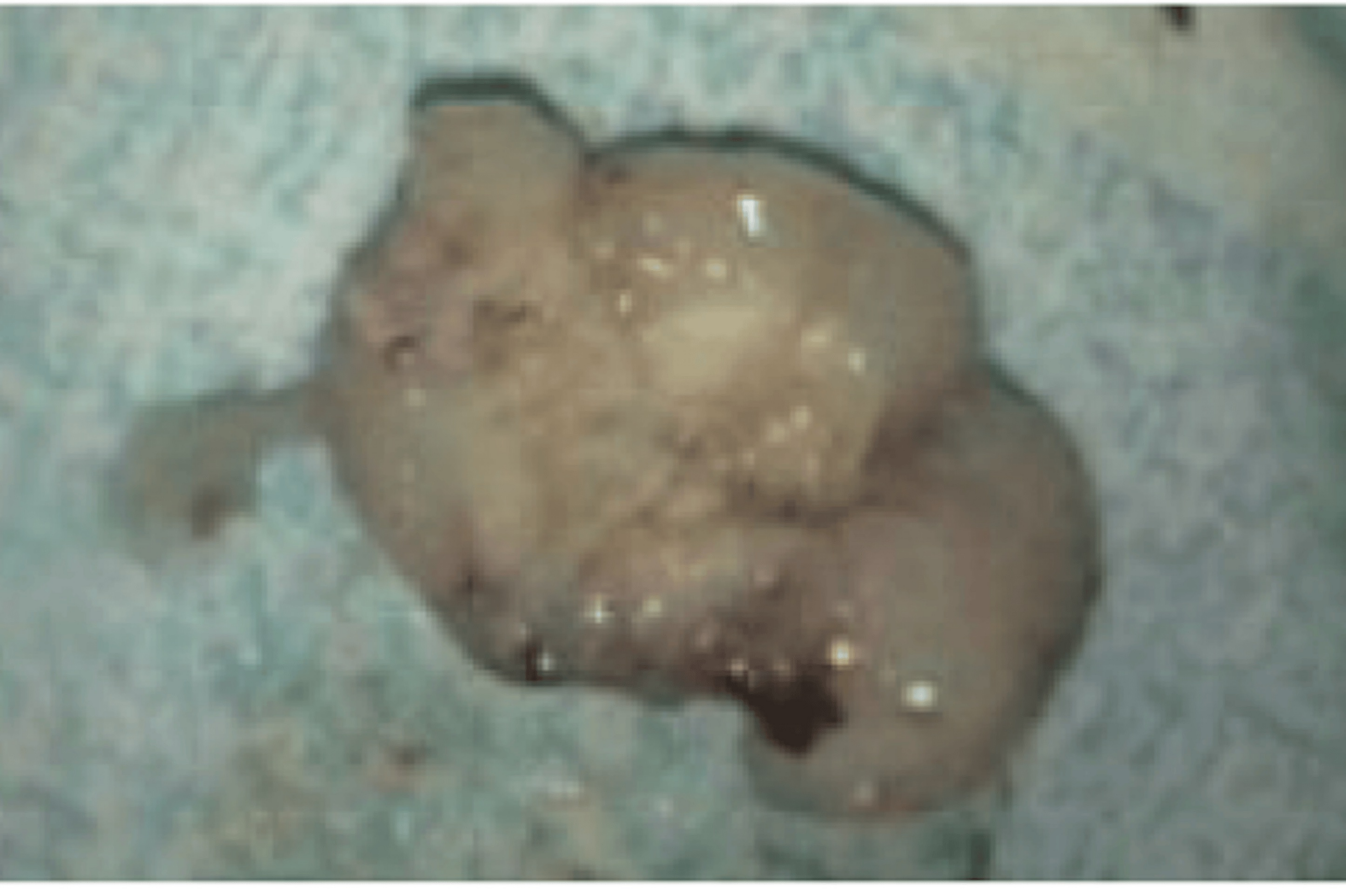
Gross specimen obtained after endoscopic snare papillectomy demonstrating the resected ampullary lesion The specimen appears nodular and polypoid with focal areas of surface hemorrhage.

During this ERCP, choledocholithiasis was identified and completely removed by balloon extraction, the previously placed CBD stent was removed, purulent drainage was noted during biliary sweeping, and snare papillectomy of the ampulla (ampullectomy) was performed. Figure 5 demonstrates an ampullary mass specimen. Following resection, a temporary pancreatic duct stent and a plastic CBD stent were placed. The patient was referred for surgical evaluation for possible pancreaticoduodenectomy (Whipple procedure) due to concern for underlying malignancy in the setting of positive margins.

Approximately one month after the second ERCP and ampullectomy, the patient re-presented with abdominal pain, nausea, and vomiting. Repeat ERCP showed a stenosed papillary orifice, and both CBD and pancreatic duct stents were placed; biopsy was not performed at that time due to the patient being on anticoagulation. She was advised to undergo repeat EUS and ERCP in a few weeks for repeat biopsy and further evaluation.

## Discussion

Ampullary adenomas are often identified incidentally, as most patients have no symptoms. Neoplasms of the ampulla of Vater are uncommon, with an estimated incidence of fewer than one per 100,000 individuals annually [1,2]. When symptomatic, they typically reflect biliary or pancreatic duct obstruction, most commonly presenting with jaundice or epigastric pain. Less frequent features include nonspecific gastrointestinal symptoms, abdominal discomfort, pruritus, or cholangitis [1]. In our case, the presentation was atypical, beginning with pruritus and RUQ pain, later progressing to jaundice and rising bilirubin. This highlights the importance of a broad clinical evaluation and early use of advanced imaging to clarify the diagnosis.

Establishing an accurate diagnosis is critical to selecting the most appropriate therapeutic approach. EUS offers excellent assessment of tumor depth and can alter staging by identifying submucosal or muscular involvement, directly influencing whether endoscopic versus surgical management is pursued. When used alongside ERCP, EUS also helps define intraductal spread, further refining treatment planning [3]. In our case, EUS identified an ampullary mass measuring 22.9 × 10 mm, confined to the mucosal layer, and biopsy demonstrated low-grade dysplasia. Notably, up to three-quarters of IAPNs contain an invasive carcinoma component, which may display intestinal, pancreatobiliary, or mixed differentiation [4]. Because the invasive focus is typically small and situated deeper within the lesion, it can be missed on superficial sampling. Therefore, generous or complete histologic evaluation is essential to ensure accurate diagnosis and staging [4]. Comprehensive histologic evaluation may reveal deeper invasive components that are not captured on limited biopsies [5]. Recurrence may occur, especially in lesions with higher-grade features, and can present years after the initial resection [4].

According to the 2021 guideline from the European Society of Gastrointestinal Endoscopy (ESGE), surgery is recommended in selected situations, including the presence of a periampullary diverticulum, lesions greater than 4 cm, or intraductal extension measuring more than 20 mm [6]. Although guidance exists, the exact threshold for choosing endoscopic versus surgical therapy is still evolving. Endoscopic management is generally reserved for smaller lesions without evidence of invasive cancer, particularly when they have well-defined borders, a soft consistency, and lack ulceration [7].

Ampullary adenomas are considered premalignant lesions, with malignant components already present in nearly 15-60% of patients [1]. They may develop sporadically or in the setting of hereditary conditions such as familial adenomatous polyposis (FAP) [1]. Compared with nonampullary duodenal adenomas, ampullary adenomas carry a higher risk of malignant transformation through the adenoma-carcinoma sequence, with studies reporting progression to adenocarcinoma in approximately 30% of untreated cases being considered uncommon, accounting for roughly 0.5% of all gastrointestinal malignancies [1,8]. Beyond cancer progression, if left untreated, complications due to obstruction of the biliary or pancreatic ducts, such as obstructive jaundice, cholangitis, and recurrent pancreatitis, may happen.

## Conclusions

Given the wide spectrum of clinical presentations and the potential for high-grade malignant transformation, it is essential to consider complete resection when appropriate, allowing comparison of superficial biopsy findings. In our case, initial biopsy suggested a noninvasive lesion; however, definitive resection revealed high-grade dysplasia with positive margins, raising concern for an underlying malignancy not captured on limited sampling. This underscores the importance of complete resection and thorough histologic evaluation. Although limited by the nature of a single-case report, this case highlights an uncommon presentation of ampullary adenoma and important diagnostic considerations for clinical practice. Furthermore, it illustrates the need for close, long-term surveillance given the risk of recurrence and potential progression.
